# Semi-Mechanistic Model for the Antitumor Response of a Combination Cocktail of Immuno-Modulators in Non-Inflamed (Cold) Tumors

**DOI:** 10.3390/cancers13205049

**Published:** 2021-10-09

**Authors:** Aymara Sancho-Araiz, Sara Zalba, María J. Garrido, Pedro Berraondo, Brian Topp, Dinesh de Alwis, Zinnia P. Parra-Guillen, Víctor Mangas-Sanjuan, Iñaki F. Trocóniz

**Affiliations:** 1 Department of Pharmaceutical Technology and Chemistry, School of Pharmacy and Nutrition, University of Navarra, 31008 Pamplona, Spain; aaraizsanch@unav.es (A.S.-A.); szalbaot@unav.es (S.Z.); mgarrido@unav.es (M.J.G.); zparra@unav.es (Z.P.P.-G.); 2 Navarra Institute for Health Research (IdiSNA), 31008 Pamplona, Spain; pberraondol@unav.es; 3 Program of Immunology and Immunotherapy, CIMA Universidad de Navarra, 31008 Pamplona, Spain; 4 Centro de Investigación Biomédica en Red de Cáncer (CIBERONC), 28029 Madrid, Spain; 5 Quantitative Pharmacology and Pharmacometrics, Merck & Co., Inc., Kenilworth, NJ 07033, USA; brian.topp@merck.com (B.T.); dinesh.de.alwis@merck.com (D.d.A.); 6 Department of Pharmacy Technology and Parasitology, Faculty of Pharmacy, University of Valencia, 46100 Valencia, Spain; victor.mangas@uv.es; 7 Interuniversity Institute of Recognition Research Molecular and Technological Development, Polytechnic University of Valencia-University of Valencia, 46100 Valencia, Spain

**Keywords:** cold tumors, immuno-oncology, preclinical, drug development, combination of therapeutics, mechanistic modeling

## Abstract

**Simple Summary:**

The clinical efficacy of immunotherapies when treating cold tumors is still low, and different treatment combinations are needed when dealing with this challenging scenario. In this work, a middle-out strategy was followed to develop a model describing the antitumor efficacy of different immune-modulator combinations, including an antigen, a toll-like receptor-3 agonist, and an immune checkpoint inhibitor in mice treated with non-inflamed tumor cells. Our results support that clinical response requires antigen-presenting cell activation and also relies on the amount of CD8 T cells and tumor resistance mechanisms present. This mathematical model is a very useful platform to evaluate different immuno-oncology combinations in both preclinical and clinical settings.

**Abstract:**

Immune checkpoint inhibitors, administered as single agents, have demonstrated clinical efficacy. However, when treating cold tumors, different combination strategies are needed. This work aims to develop a semi-mechanistic model describing the antitumor efficacy of immunotherapy combinations in cold tumors. Tumor size of mice treated with TC-1/A9 non-inflamed tumors and the drug effects of an antigen, a toll-like receptor-3 agonist (PIC), and an immune checkpoint inhibitor (anti-programmed cell death 1 antibody) were modeled using Monolix and following a middle-out strategy. Tumor growth was best characterized by an exponential model with an estimated initial tumor size of 19.5 mm^3^ and a doubling time of 3.6 days. In the treatment groups, contrary to the lack of response observed in monotherapy, combinations including the antigen were able to induce an antitumor response. The final model successfully captured the 23% increase in the probability of cure from bi-therapy to triple-therapy. Moreover, our work supports that CD8^+^ T lymphocytes and resistance mechanisms are strongly related to the clinical outcome. The activation of antigen-presenting cells might be needed to achieve an antitumor response in reduced immunogenic tumors when combined with other immunotherapies. These models can be used as a platform to evaluate different immuno-oncology combinations in preclinical and clinical scenarios.

## 1. Introduction

Immuno-oncology (IO), or cancer immunotherapy, focuses on the ability of the immune system to detect and eliminate cancer cells [1]. In this regard, recent development in checkpoint inhibition therapies, tumor-infiltrating lymphocytes therapies, chimeric antigen receptor T cell therapies, and cancer vaccines have led to significant advances in cancer treatment [2]. Although the different immunotherapy approaches have diverse ways of modulating the natural defenses, tumor cells are still able to evade the immune system and proliferate over time [1]. Some of the resistance mechanisms developed by tumors include myeloid-derived suppressor cells (MDSCs), regulatory T cells (Tregs), and immunosuppressive cytokines and inhibitory proteins, such as transforming growth factor-β (TGF-β), interleukin (IL)-4, programmed cell death 1 (PD-1), and programmed cell death ligand 1 (PD-L1) [3].

Bearing this in mind, immune-checkpoint inhibitors (ICIs) targeting cytotoxic T-lymphocyte antigen-4 (CTLA-4), PD-1, or PD-L1, have generated substantial interest [4]. These therapies are characterized by their long-lasting antitumor responses compared to conventional treatments [5,6]. Different monoclonal antibodies that target PD-1 have shown an objective response rate of 40–45% for the treatment of non-small cell lung cancer (NSCLC) [7]. However, in other cancer types, only a fraction of patients benefit from these therapies. For instance, in triple-negative breast cancer, a modest response rate of 20% has been shown [8]. With this wide range of responses, there is an increasing interest in discovering tumor microenvironment predictive biomarkers [7]. The success of ICI monotherapies has been associated, in part, with tumor immunogenicity, the amount of T-cells infiltration, and the expression of PD-L1 [9,10]. According to the extent of immune cell infiltration, among other factors, tumors can be classified as immune desert, immune excluded, and immune inflamed. Immune desert tumors, also known as cold tumors and associated with resistance phenotypes, are characterized by the absence of T-cells infiltrated in the tumor microenvironment, low tumor mutational burden, low expression of PD-L1, and poor antigen presentation [9].

Treating cold tumors can be very challenging. Nevertheless, tumor inflammation, which allows tumor-specific T-cell trafficking, has been identified as a key goal of the immunomodulatory approaches for these tumors [11]. One of the proposed approaches to overcome the lack of preexisting immune response consists of combining a priming therapy that enhances T cells responses (such as vaccines or adoptive T cell transfer (ACT)), with the removal of co-inhibitory signals (through approaches such as ICI or MDSC depletion) and/or the supply of co-stimulatory signals [9,11]. Therefore, it is important to develop combination strategies [2,10].

The combination of ICIs with vaccines or toll-like receptor (TLR) agonists has proven to enhance treatment efficacy in preclinical and clinical scenarios [10,12,13,14]. A recent work carried out in mice [15], showed that triple therapy, including a peptide, a TLR, and an ICI, was the most efficient strategy at remodeling myeloid cells and inducing antitumor immunity. In a phase Ib trial in pancreatic patients, the combination of anti-CTLA-4 (ipilimumab) with GVAX vaccine improved overall survival (OS) compared to ipilimumab monotherapy [14]. In a different clinical trial, HPV-16-positive cancer patients were vaccinated with a synthetic long-peptide vaccine in combination with nivolumab with an overall response rate of 33% compared to 16–22% when administered nivolumab alone [16].

The use of peptide-based vaccines is a widely studied approach that increases the number and/or availability of antigen-specific T cells by activating professional antigen-presenting cells (APCs) such as dendritic cells and that can be used as a priming therapy [17]. In addition, toll-like receptors (TLR), for instance, can be used to activate the innate immune system and enhance adaptive immune responses [13,18]. Among TLR ligands, different response patterns can be observed [19], and thus, the selection of a particular type matters. Polyinosinic-polycytidylic acid (Poly(I:C)), a synthetic TLR3 agonists, administered together with peptide vaccination, has been demonstrated to be a viable strategy resulting in enhanced proliferative and functional immune response, ultimately resulting in antitumor immunity [19].

Although there have been great advances in IO, there are still many unanswered questions about how the immune systems interact with a growing tumor and which components of the immune system play significant roles in responding to immunotherapy. In this sense, mathematical modeling provides a useful tool to better understand complex systems, and identify mechanisms that could explain the observed clinical or preclinical outputs [20].

With the recent eruption of quantitative system pharmacology (QSP) models in the IO field, a high degree of granularity of the biological system has been achieved. Examples include monotherapy and combination scenarios, including nivolumab therapy in NSCLC [21], HER2-negative breast cancer patients treated with anti-PD1, entinostat, and anti-CTLA-4 [22], or administration of a vaccine, anti-IL2, and ICIs in prostate cancer patients [23]. Those models, which are fed with several parameter values obtained from different data sources, including in vitro and preclinical in vivo experiments, are able to reproduce adequately the response rates obtained in the clinical trials. However, their inherent complexity prevents the estimation of a patients’ specific parameters and covariates identification.

In this regard, the middle-out modeling approach, in which the known biological processes and pharmacodynamic mechanisms are included while keeping model and model parameters identifiable, provides a quantitative platform for model development in preclinical and clinical scenarios. A semi-mechanistic pharmacodynamic model was previously developed for the antitumor effects of the combination between an antigen, a toll-like receptor agonist, and a chemotherapy agent in a hot tumor mice model [12,24]. In addition, Kosinsky et al. [25] developed a model capable of reproducing the dynamics of a hot tumor in mice after the combined administration of radiation and anti-PD-L1. Regarding cold tumors, there is a scarcity of mathematical models describing both tumor dynamics and/or exploring possible predictive biomarkers.

In the current work, we adapted and expanded the former computational work [12] to cold tumors, mapping the impact of the myeloid cells and regulatory mechanisms on CD8^+^ T cell activation, expansion, and exhaustion. As a complementary approach, this work aims to develop a semi-mechanistic pharmacodynamic population model describing longitudinal tumor size data gathered in a mice model of cold tumors where different IO combinations are administered.

## 2. Materials and Methods

### 2.1. Study Design

Data gathered from a total of 121 (5-weeks old) immunocompetent C57BL/6J female mice were used in the current analysis [15]. Tumor cells (1 × 10^5^ TC-1/A9) expressing HPV E7 protein were inoculated subcutaneously on the right flank (day 0). The cell line used in the experiments was derived from primary mouse lung epithelial cells, expressed the E7 protein from HPV, and was characterized by a low expression of major histocompatibility complex class I (MHC I) [15]. Additionally, previous experiments [15] demonstrated that this cell line presented very low tumor-infiltrated lymphocytes (TILs) and low expression of PD-L1. Considering this information, the TC-1/A9 tumors may be considered a non-inflamed or cold tumor model.

When the average tumor diameter reached 5 mm, mice were randomly divided into groups receiving treatments consisting of mono-, bi-, or triple-therapy based on an E7 long peptide (Antigen (Ag)), a TLR-3 agonist (polyinosinic-polycytidylic acid (PIC)) (ThermoFisher; Massachusetts MA, USA), and an anti-PD1 (αPD1) (CD279; clone RMP1-14; BioXCell; New Hampshire NH, USA). Table 1 shows the different treatment groups with the corresponding number of mice. Ag and PIC were administered intratumorally at days 7 and 14, whereas αPD1 was injected as an intravenous bolus at days 7, 10, and 14 after tumor implantation. One group of animals received an additional dose of αPD1 on day 17. Additional details of the experimental conditions can be found elsewhere [15].

Tumor size (TS) measurements were monitored twice a week until mice reached the maximum TS allowed according to European animal care regulation and to the protocol approved by the Ethics Committee of the University of Navarra (Ref. 023-17). Tumor volume was calculated, as shown in Equation (1), assuming that the tumor has an ovoid form [26].

(1) $$Tumor\;Size\;Volume=Length\times \frac{Width^{2}}{2}$$

Animals presenting TS shrinkage were monitored for three months in order to detect possible tumor relapse.

### 2.2. General Description of the Data

**Figure 1:**
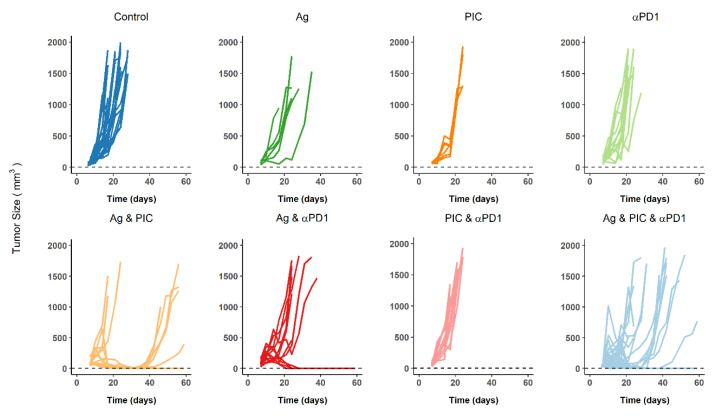
Individual raw data profiles. Tumor size over time, computed as volume based on two perpendicular diameters, is represented for each mouse and each treatment group included in the analysis. Horizontal dashed lines represent the lower limit of quantification (4 mm^3^).

Tumor size profiles for the 8 different treatment groups ((i) control, (ii) Ag, (iii) PIC, (iv) αPD1, (v) Ag and PIC, (vi) Ag and αPD1, (vii) PIC and αPD1, (viii) Ag and PIC and αPD1) are presented in Figure 1.

The exploratory analysis of the data yielded the following processes which were considered during model development: (i) tumors grow exponentially in the absence of treatment administration, (ii) Ag administration is crucial to trigger a therapeutic effect [13], (iii) TS shrinkage is not observed with any of the treatments in monotherapy, nor with PIC and αPD1 bi-therapy treatment, and (iv) mice receiving bi-therapy of Ag and PIC, or Ag and αPD1, or triple-therapy (Ag and PIC and αPD1) showed a tumor response associated with a wide heterogeneity and thus, mice were classified as responders (TS below the limit of quantification at the end of the study), non-responders (TS profiles similar to those of the control group), or partial responders (mice able to trigger just a transient response).

### 2.3. Data Analysis

TS data were logarithmically transformed for the analysis performed in MONOLIX (nonlinear mixed effects modeling) Suite 2019R2 [27]. Parameters were estimated by computing the maximum likelihood estimator of the parameter using the stochastic approximation expectation–maximization algorithm combined with a Markov chain Monte Carlo procedure. For graphical output and statistical analysis, the R software [28] (version 4.0.4) was used. Model development was carried out with data from control, monotherapy, and bi-therapy groups of Ag and PIC and Ag and αPD1, whereas data from the triple-therapy and bi-therapy of PIC and αPD1 were used for external validation.

TS measurements equal or lower than 4 mm^3^, considered as the low limit of quantification, represented approximately 33% of the total and were thus, considered as censored information and modeled based on the M3 method [29]. Inter-animal variability (IAV) was modeled exponentially. Residual error was described with an additive model in the logarithmic domain.

#### 2.3.1. Model Selection

Selection between models was based on the minimum value of the objective function provided by Monolix, which is equal to −2 × log-likelihood (−2LL), the precision of parameters estimates, and the visual exploration of goodness-of-fit plots. For nested models differing in one parameter, the application of the −2LL ratio test differences of 3.84 were considered significant at the 5% level.

#### 2.3.2. Model Evaluation and Validation

Model evaluation and validation were performed through numerical predictive checks. For each study, 1000 simulated datasets were generated using inter-animal and residual variability. The probability of cure—the ratio between the number of responders and the total number of simulated mice—and the probability of partial response—the ratio between the number of partial responders and the total number of simulated mice—was obtained for each treatment group and each simulated dataset. Then, the 5th, 50th, and 95th percentile were computed and compared with the raw data. Additionally, parameter precision was evaluated from the analysis of 1000 simulated bootstrap datasets.

#### 2.3.3. Model Building

**Figure 2:**
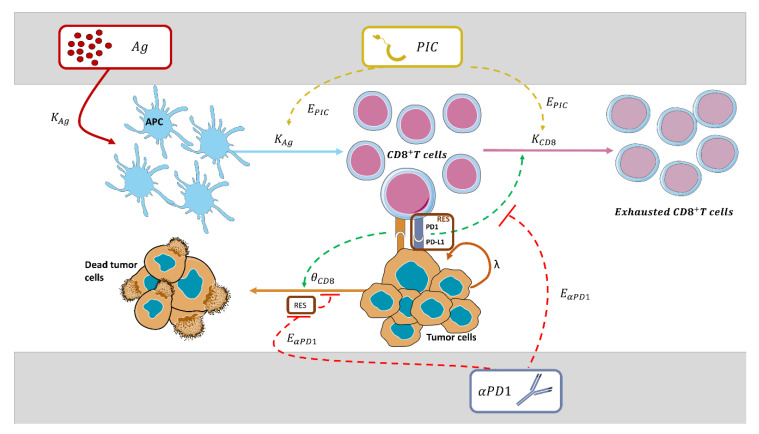
Schematic representation of the immuno-oncology therapy model, including the interactions of immune cells with cancer cells after the administration of antigen Ag, PIC, and αPD1. Dashed green arrows indicate activation, dashed red blocked arrows indicate inhibition, dashed yellow arrows indicate the effect of PIC, and solid sharped arrows indicate the transit between compartments. TRT and TR denote treatment and transit compartments, respectively. APC stands for antigen-presenting cells, and RES for the resistance mechanisms developed by the tumor. A description of the parameters can be found in Material and Methods.

Model development was performed sequentially and following a middle-out approach because we not only had one response variable, which was tumor size, but also because not all the treatment groups were informative with respect to certain mechanisms (i.e., bi-therapy between Ag and PIC, or Ag and aPD1). The final model structure was based on the experimental data and current knowledge of the system. Table 2 provides an overview of the biological processes included in the model (Biological Processes), the experimental data supporting those processes (Experimental data) and the different model assumptions for each step of model building (Model assumptions). Additionally, a schematic representation of the dynamics of immune cells, their role in tumor response, and the mechanisms of the different IO treatments are shown in Figure 2.

#### 2.3.4. Model for Unperturbed Tumor Growth

The model describing unperturbed tumor growth was developed using only data from the control group (in the absence of any therapeutic agent). Different models, including linear, exponential growth, and Gompertz [41,42], were evaluated to describe the continuous TS growth depicted in Figure 1. Tumor growth was described by the exponential model (Equation (2)), where λ (first-order rate constant) should be interpreted as the difference between cell proliferation and degradation. The initial condition of the tumor system is TS_0_, the value of TS at the time of cell inoculation.

(2) $$\frac{dTS}{dT}=\lambda \times \mathrm{TS}\;$$

#### 2.3.5. K-PD Models

To generate the signals triggered by Ag, PIC, and αPD1 administration, a cascade of transit compartments for the Ag, PIC, and αPD1, was implemented as follows:

(3) $$\frac{dTRT_{i}}{dt}=-K_{i}\times TRT_{i}$$

(4) $$\frac{dTR_{i}}{dt}=K_{i}\times TRT_{i}-K_{i}\times TR_{i}$$

where *K* is a first-order rate constant, and *TRT_i_* and *TR_i_* stand for treatment and transit compartments from where drug effects are elicited, respectively, and where *i* takes the form of Ag, PIC, and αPD1. For the three therapies, the initial conditions for *TRT_i_* and *TR_i_* were 1 and 0, respectively. From a modeling point of view, the inclusion of transit compartments allows predicting a treatment response with a certain delay regarding administration time. Delayed responses are quite common (they represent the rule rather than the exception) and can be explained by a great variety of possible mechanisms. In the current model, the Ag transit compartment (*TR_Ag_*) might represent the APCs (Table 2). Nevertheless, TR_PIC_, and TR_αPD1_ could reflect different drug distribution into the target compartment as well as tumor infiltration mechanisms (Table 2).

#### 2.3.6. Model for CD8 Activation, Expansion, and Tumor Response

As it has been depicted in Table 2, in this model, the tumor cell death is promoted by the activated CD8^+^ T cells (*CD8_act_*) yielded by Ag administration (*TR_Ag_*), which were further expanded by the presence of PIC.

(5) $$\frac{dCD8_{act}}{dt}=K_{CD8}\times TR_{Ag}-K_{CD8}\times CD8_{act}\;$$

(6) $$\frac{\mathrm{dTS}}{dt}=\lambda \times \mathrm{TS}-\theta_{CD8}\times CD8_{act}\times \mathrm{TS}$$

where *K_CD8_* is a first-order rate constant for CD8 activation, constrained to have the same value as *K_Ag_* to ensure parameter identifiability, and θ*_CD8_* is a second-order rate constant representing the efficiency of CD8^+^ T cells to promote tumor cells death. The initial condition for *CD8_act_* was set equal to zero. The first term of Equation (6), which describes tumor proliferation (λ × TS), is assumed to remain unaffected upon treatment administration.

Given the fact that tumor response was obtained after Ag and PIC bi-therapy, and not with either of the two treatments given alone, the following model building strategy was used to identify the parameters: First, tumor response after bi-therapy with Ag and PIC was characterized to obtain the estimate of θ*_CD8_*, assuming the two treatments as a single active compound. Equations (5) and (6) represent the model structure used to describe *CD8_act_* and TS dynamics. In a second step, using the value θCD8 from the first step, the parameter *K_Ag_* (Equations (3) and (4)) was obtained by analyzing the subject in the Ag monotherapy group showing tumor response. Then, *K_Ag_* was adjusted and calibrated to provide *TR_Ag_* levels that would trigger insufficient *CD8_act_* to cause tumor response (resembling the case of non-responder animals, which was the main behavior in that treatment group) (Figure 1). Finally, TS profiles data after Ag and PIC bi-therapy were re-analyzed. Treating both as different therapies and using the last estimates of θ*_CD8_* and *K_Ag_*, the parameter K_PIC_ (Equations (3) and (4)) was estimated, and a model structure for the effect of TR_PIC_ on *K_Ag_*/K_CD8_ (E_PIC_) was selected.

#### 2.3.7. Model for Tumor Resistance to Treatment Effects

A resistance parameter (Resistance) was incorporated to account for regulatory T cells, MDSCs, or the expression of PD-1 (Table 2). The effect of αPD1 (E_αPD1_) led to enhanced tumor response by inhibiting the tumor Resistance, diminishing the relapse phenomena (Resistance/E_αPD1_)). Resistance was included in the model, assuming an arbitrary value of one for all mice and during the entire period of the study. Parameter K_αPD1_ (Equations (3) and (4)) and the structure of the model for the inhibitory effect of TRT_αPD1_ on Resistance were obtained from the analysis of the animals receiving bi-therapy with Ag and αPD1, and using the obtained estimates of θ*_CD8_* and *K_Ag_* described above.

### 2.4. Model Exploration

During the model exploration, simulations were performed in order to evaluate the impact of the amount of *CD8_act_* and the Resistance on response rates. For K_PIC_ and K_αPD1_, a sequence of 10 values inside the 90% confidence interval was obtained for each of them and subsequently combined to create 100 different combinations while the rest of the parameters were fixed to the final estimates of the selected model. Then, for each parameter and treatment combination, one thousand TS profiles were simulated. The percentage of response status—responders, non-responders, and partial responders—was calculated together with the area under the curve (AUC) of *CD8_act_* and resistance inhibition induced by αPD1 time profiles (AUC_CD8_ and AUC_resistance inhibition_, respectively).

## 3. Results

### 3.1. Mathematical Model

Among the different models explored to describe tumor growth in the absence of treatment administration, TS profiles were best characterized by exponential growth, assuming that the growth rate is proportional to the tumor burden. The model provided estimates for TS_0_ and λ of 19.5 mm^3^ and 0.194 days^−1^ (time for doubling the size of the tumor is 3.6 days), respectively. IAV was found significant (*p* < 0.01) with a low magnitude estimate (13.5%) for λ, but not significant for TS_0_ (*p* > 0.05).

Once the model for tumor progression was established, the parameters for the APC activation by the Ag and CD8^+^ T cell activation/expansion by PIC were identified. The analysis of the group of mice receiving Ag and PIC but treated as monotherapy (Equations (3)–(6)) yielded an estimate of θ*_CD8_* of 1.63 au × days^−1^, with an estimate of IAV of 46%.

In the second step, using the estimate of θ*_CD8_*, the value of *K_Ag_* obtained from the single animal showing tumor response after administering Ag in monotherapy was 0.931 days^−1^. The fine-tuning analysis revealed that a value of *K_Ag_* of 4.93 days^−1^ was high enough to trigger a fast elimination of Ag and thus, suppress its induced response. The model adequately described the data, but the number of transit compartments could not be estimated with precision. Consequently, one intermediate (transit) compartment was introduced at a time, and the AIC and model performance was compared for the different number of compartments. In this case, one transit compartment (TR_AG_) where the amount of signal (APCs) would be responsible for inducing CD8 cell activation proved to be enough to describe the data successfully (ΔAIC of −12.47, −0.5, and −1.65 points compared with no delay, two or three transit compartments, respectively).

Following model building, therapy of Ag and PIC was re-analyzed using the above new estimates of θ*_CD8_* and *K_Ag_* to isolate now the contribution of PIC to CD8 activation and expansion and subsequent tumor response. Linear and nonlinear (i.e., E_max_, sigmoidal E_max_) drug exposure-effect relationships were explored for these processes. In this case, the E_PIC_ was best characterized by a linear model with the structure of 1/(1 + θ_PIC_ × TR_PIC_). This effect enabled to decrease APCs elimination (Equation (7)) and sustain the amount of *CD8_act_* present in the system by modulating both the proliferation and elimination processes (Equation (8)). Similar to the Ag, we could not estimate the number of transit compartments for PIC with precision either. In this case, the data was best described with one transit compartment. Additionally, it was found that the estimate of K_PIC_ was ~7 times lower than *K_Ag_*.

Finally, αPD1 elicited its effects (E_αPD1_), decreasing the mechanisms of *CD8_act_* exhaustion (Resistance, Equation (8)) and enhancing *CD8_act_* efficacy to induce tumor death (Equation (9)). The structure selected for E_αPD1_ had the form of (1 + θ_αPD1_ × TR_αPD1_). For αPD1, an additional transit compartment was introduced (ΔAIC of −653.58, −91.71, and −13.29 points compared with no delay, one or three transit compartments, respectively) (Equation (4)). K_αPD1_ was estimated with a value of 2.3 × 10^−3^ days^−1^ and the estimate of θ_αPD1_ resulted 68% lower than the one obtained for θ_PIC_.

Equation (7) illustrates the Ag transit compartment (*TR_Ag_*) (Equation (4)), representing the APC activation by the Ag, and Equations (8) and (9) generalize expressions 5 and 6 respectively to account for the PIC and αPD1 effects.

(7) $$\frac{dAPC}{dt}=K_{Ag}\times TRT_{Ag}-K_{Ag}\times APC\times E_{PIC}$$

(8) $$\frac{dCD8_{act}}{dt}=K_{CD8}\times APC\times E_{PIC}-K_{CD8}\times CD8_{act}\times E_{\mathrm{PIC}}\times \frac{Resistance}{E_{\alpha \mathrm{PD}1}}$$

(9) dTSdt=λ×TS−θCD8×CD8act(ResistanceEαPD1)×TS

**Figure 3:**
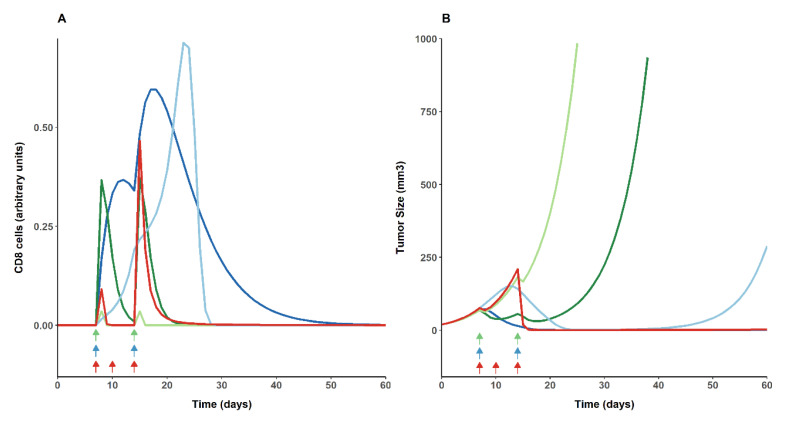
The typical model-predicted profiles of activated CD8 cells (**A**) and tumor size (TS) (**B**) over time, corresponding to the different treatment groups: antigen monotherapy for responder (dark green) and non-responder mice (light green), Ag and PIC treated as monotherapy (dark blue) and as bi-therapy (light blue), and Ag and αPD1 (red). Arrows indicate the day of dosing time for antigen (green), PIC (blue), and αPD1 (red).

Figure 3 shows the typical profiles of the resulting CD8 (3A) and TS responses (3B) response for the typical parameters in the different steps of the model building. For the responder mice in the Ag monotherapy (dark green), the amount of CD8 cells exceeds 0.25 arbitrary units, which is reflected in a delayed tumor growth. However, for non-responder mice (light green) it can be observed the profile of CD8 dynamics eliciting an inefficient tumor response.

Moreover, the figure illustrates the effect of both PIC and αPD1 in combination with the Ag. The administration of PIC (dark and light blue), delayed the turnover of both TR_AG_ and CD8 cells (Equations (7) and (8)), increasing the levels of *CD8_act_* and inhibiting tumor growth dynamics. Despite this, when Ag and PIC are treated as a bi-therapy (light blue), a relapse at around day 40, similarly to the raw data (Figure 1), can be observed. Furthermore, Figure 3 shows in red the profiles after the administration of PIC and αPD1. The CD8 cells increase remarkably after the second dose of αPD1, and the tumor growth is inhibited without showing relapse.

Table 3 lists the final parameter estimates of the model. All parameters were estimated with acceptable precision as reflected by the relative standard errors, which assume symmetric distribution of the confidence intervals and the non-parametric bootstrap analysis results. Note that precision associated with the parameter K_AG_ is not provided as the corresponding estimate was obtained through a fine-tuning exercise, as described in the methodology section. The magnitude of IAV associated with K_PIC_ and K_αPD1_ exceeds 100% resembling the heterogeneity seen in the TS profiles in all the treatment groups (Figure 1) in which a certain degree of response was achieved (responders, partial responders, and non-responders).

**Figure 4:**
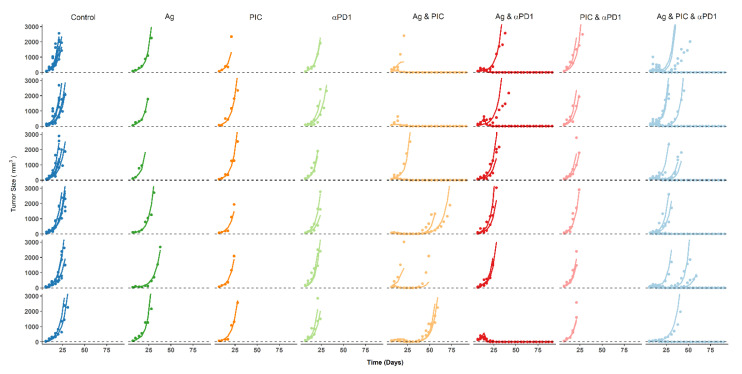
Individual model predictions. Tumor size observations (points) and individual model predictions (lines) for all the treatment groups included in the analysis. 4 mm^3^ was considered as the limit of quantification (dashed lines). For those treatment groups with a higher number of animals, more than one TS profile was included in each panel.

The value of ε-shrinkage was 15%. Therefore, individual predictions can be considered informative for the evaluation of model performance. Observed and predicted individual profiles are shown superimposed in Figure 4, where an excellent agreement can be observed for the vast majority of the mice between observations and predictions, not only for those treatment groups used during model development but also for the triple therapy (validation treatment group).

### 3.2. Model Evaluation and Validation

**Figure 5:**
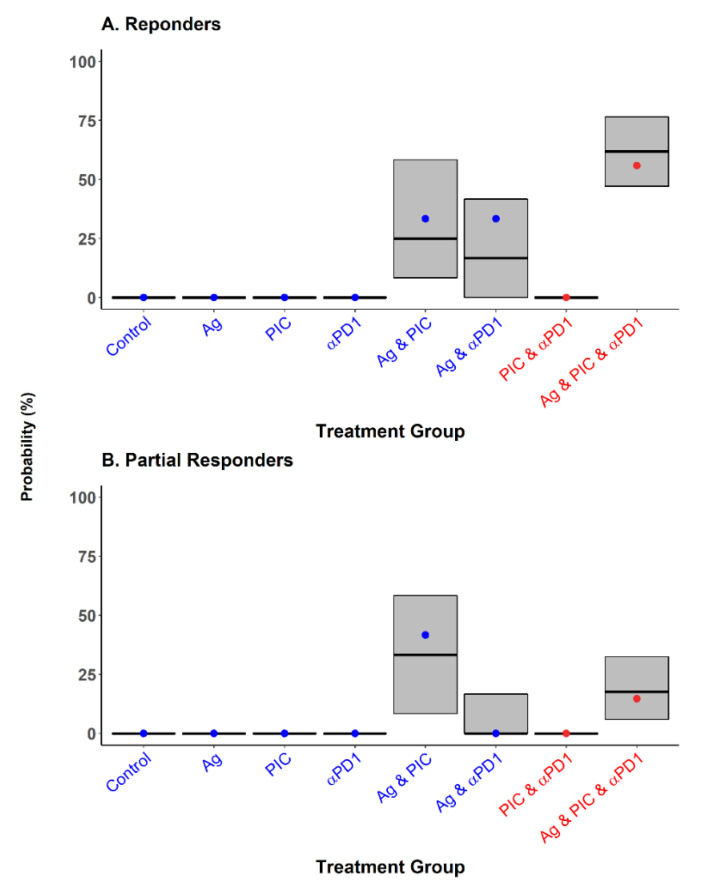
Simulation-based model diagnostics. The percentage of cure (**A**) and partial response (**B**) calculated over 1000 simulated studies is presented and compared with the percentage calculated from the raw data. Grey areas represent 90% prediction interval of the simulated data, with the horizontal line in black showing the median of the distribution. Dots correspond to the percentages obtained from the observed data (blue for the treatment groups used for model development and red for the triple-therapy group used for external validation).

Figure 5 explores model performance through simulation-based diagnostics using the probability of responder and partial responder animals within each treatment group as the metrics of interest. The metrics computed from the raw data fall close to the median derived from the simulated studies for all treatment groups. Overall simulated data resembled the percentage of response found in the raw data for each treatment group. Remarkably, the model was able to adequately capture the 23% increase in the probability of cure in the group of animals administered with triple-therapy compared to the bi-therapies of Ag and PIC and Ag and αPD1.

### 3.3. Model Exploration

**Figure 6:**
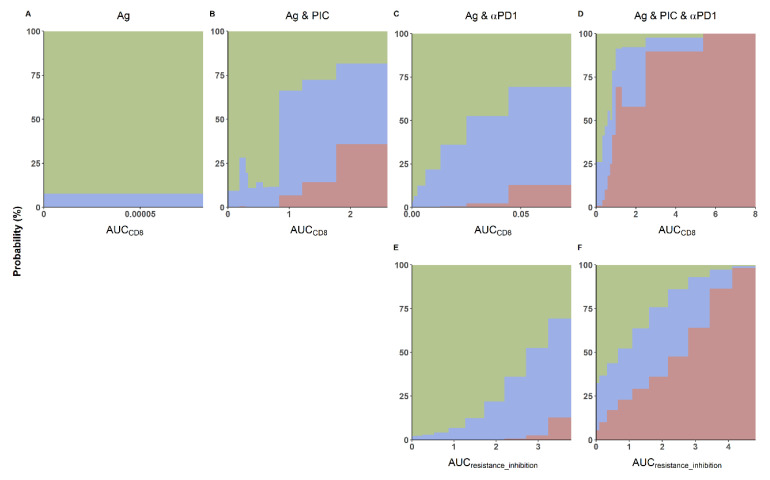
Effect of the area under the levels of activated CD8 (**A**–**D**) and resistance inhibition induced by αPD1 (**E**,**F**) vs. time curve (AUC_CD8_ and AUC_resistance_inhibition_, respectively) on response rates expressed as a percentage of mice showing total response (red), transient response (blue), and absence of response (green). One thousand animals for each of the 100 different parameter combinations were simulated for the multiple treatment groups: (**A**) Ag, (**B**) Ag and PIC, (**C**,**E**) Ag and αPD1, and (**D**,**F**) triple-therapy.

Systematically, results indicated a strong relationship between the degree of response (represented in this exercise as a percentage of animals responding, non-responding, or with a partial response to the treatment) and the exposure of CD8 cells (AUC_CD8_) or the different resistance mechanisms (AUC_resistance inhibition_). In Figure 6A,C, it can be observed that the AUC_CD8_ does not exceed 1 (in arbitrary units) when vaccine is administered alone or in combination with aPD1, and that none or very low percentage of cured mice is reached. The co-administration of the Ag with PIC (Figure 6B) is needed to achieve at least 2 arbitrary units of AUC_CD8_, which will provide at least a probability of complete response of 25%. Similar findings can be found in Figure 3, in which also, the higher exposure of CD8 cells is observed in the bi-therapy of Ag and PIC.

Results presented in the lower panels of Figure 6E,F suggest that inhibition of the tumor-related resistance mechanisms, represented by AUC_resistance inhibition_, is less efficient with respect to antitumor effects than CD8 proliferation and expansion. The bi-therapy of Ag and PIC is able to achieve at least a response rate of 25%, while high values of AUC_resistance inhibition_ in the Ag and αPD1 treatment group do not reach 15% of the response.

The interaction between CD8 expansion and tumor resistance inhibition mechanisms appears to be synergistic as the AUC_CD8,_ and AUC_resistance inhibition_ in the triple-therapy groups (Figure 6D,F) increased beyond additivity with respect to the expectation from the two bi-therapy groups with Ag. For intermediate AUC_CD8_ values (between 1 and 2 in arbitrary units) and bi-therapies treatments, response rates below 25% is observed; whereas for the triple-therapy, between 25–70% of the animals respond to the treatment.

## 4. Discussion

In this work, we have developed a semi-mechanistic model for the antitumor effects of combination treatments based on three immune-modulators—a tumor-antigen, a toll-like receptor-3, and an αPD1—in a cold tumor. A middle-out approach was followed during model building since; (i) selection between competing models was evaluated through their ability to describe the longitudinal tumor size measurements, and (ii) model structures were constrained to resemble the current knowledge of the systems [12,43,44]. The mechanisms of action characterized for these therapies included the activation of APCs, the activation and expansion of CD8 cells, and the inhibition of tumor resistance mechanisms (Table 2). The model proposed integrates some immune components and pathways of the complex biological system, however, its development was mainly data-driven based on just a single response variable (tumor size). This fact justifies both the number of model assumptions and that the model parameters represent, in this case, several biological processes that cannot be shred. Notwithstanding this, the selected model showed very good performance at both individual and population levels with precise parameter estimates and demonstrated to be robust across the validation set of data.

Under the assumption that the lack of responses observed in a variety of cancer patients receiving ICI monotherapies [18,45] is likely due to reduced tumor immunogenicity [9,10], we have attempted to mimic the actual clinical scenario. Firstly, performing the animal experiments inoculating a cell line characterized by a cold tumor [15], and secondly, following the current treatment paradigms where a variety of combinatorial approaches are being investigated [2,4,18].

Among the three different immunotherapeutic agents included in the analysis, tumor-antigens are the main stimuli that trigger the immune response. They are able to activate APCs and then promote a T cell-mediated immune response capable of recognizing and eliminating cancer cells expressing specific tumor-associated antigens [1,13,17,35]. However, in our experiments, when Ag monotherapy is administered, all animals except one showed a complete lack of response when the Ag was administered in monotherapy (Figure 1). The possibility that those results are due to an experimental artifact and not to the cold nature of the tumor model is unlikely as the individual TS profiles are comparable to other published results [35,46], which also treated poorly immunogenic tumors with different IO combination approaches. Therefore, in the present work, the characterization of APC activation caused by the injection of the Ag in the tumor represents a modeling aspect that deserves consideration, as it allowed us to dissect quantitatively the mechanisms involving CD8 dynamics. Alternatively, a simpler approach would have been to treat the combination of Ag and PIC as a monotherapy (as we did in the previous modeling steps) and describing the result of both effects together. The additional efforts here performed to isolate the individual drug contributions and mechanistically characterize their interactions allowed us to suggest that APC activation is required for PIC to trigger CD8 activation and expansion. This might have important implications as, based on our results, APC activation is required to achieve a tumor response and might be a key element involved in turning a cold tumor into hot tumor [9,11].

As regards the exacerbation and maintenance of the Ag effects by PIC, our result can be compared with previous experiments [10,13,18]. Zalba et al. [15] found similar kinetics of CD8^+^ T lymphocytes when E7 long peptide and PIC were administered in combination compared to typical model-predicted profiles of activated CD8 cells (Figure 3). Moreover, we compared our findings with our previous work [12] in which an antigen, a toll-like receptor 9 (TLR-9), and a chemotherapeutic agent were administered in combinations to a murine hot tumor model. Using the past model and the corresponding parameter estimates, the impact of the TLR agonist over the effector cells was calculated and compared to the results obtained in the current analysis summarized in the typical activated CD8 cells profiles represented in Figure 3. The TLR-9 was able to double and accelerate the immune response elicited by the antigen when both agents were administered in combination. Conversely, in the present work, when Ag and PIC were administered to a cold tumor, a larger increase and delayed in time was observed. The discrepancies might be explained due to the different response patterns observed across the TLR ligands [19]. In particular, the TLR-9 agonists are known to induce a faster response to peptide administration [47], whereas PIC enhances the expansion and delays the contraction of antigen-specific CD8^+^ T cells [19].

Lastly, the inclusion of αPD1 effects entailed also the inclusion of tumor resistance. Different mechanisms have previously been incorporated in mathematical models, such as a regulatory compartment controlled by tumor size [12], the proliferation and accumulation of immune-suppressive cells (Tregs), and the PD-1/PD-L1 expression on cancer cells [21,25]. In the actual work, contrary to other models developed using data of experiments in which treatments were administered at different times after cell inoculation [12,24], the three therapeutic agents are always administered at the same time. This fact hinders the development of a time-variant model for tumor resistance. For this reason and given the fact that a temporal or complete lack of tumor response is observed for some animals in every treatment (Figure 1), the magnitude of the tumor resistance is considered to remain constant through all the experimental period and only decreased under the presence of αPD1. A recent work developed by Jafarnejad et al. [21] showed a flat profile of CD4^+^ T cells up to 3 months for the different responses (complete or partial response, stable disease, or progressive disease), suggesting that our assumption is reasonable. This QSP model also incorporated the effects of an αPD1, nivolumab, as the binding of the agent and the blocking of the PD-1 receptor on the surface of T cells (CD8 cells and regulatory T cells). A later model expansion [25] also included the expression of PD-L1 expression in antigen-presenting cells [39]. In a different work [25], the ICI was similarly able to inhibit the PD-L1 expression and thus, suppress the negative feedback that facilitated the process of effector T cells exhaustion and apoptosis. The analysis revealed that 250 effector T cells/µL were reached when mice were treated with radiation monotherapy, but the administration of an αPD-L1 concurrently with radiation was capable of increasing this amount to a maximum of 700 effector T cells/µL.

In both preclinical [12,13,25] and clinical [7,8,21,22], a high degree of variability in response to cancer treatments is observed. Similarly, our experiments show that, despite the success of the triple-therapy (Ag, PIC, and anti-PD1), there is still a wide response heterogeneity (Figure 1). Previous works [12,24,25] described differences between responders, non-responders, and partial responders with the use of mixture models which allocated individuals to different subject populations independently from specific covariates/biomarker indicators. In contrast, the current work handles such differences between tumor size profiles using unimodal distributions of random effects. Consequently, the high heterogeneity seen in the tumor size profiles of the mice under treatment is reflected in the magnitude of variability estimated for certain parameters of the model.

As has been noted, a deep understanding of immune response dynamics and the interplay with tumor-infiltrating processes and tumor cell growth can help to overcome the current challenges in IO and, in this sense, the mathematical model plays an important role. Several QSP models have been published for single agent or combination therapies and applied to different types of tumors, including melanoma, breast cancer, and NSCLC [21,23,25,40]. However, the development of these types of models is challenging due to the large number of parameters, many of them taken from different sources, and the relatively small amount of observed data usually available. In this respect, the type of model developed in the current work is complementary to the QSP models as it fulfills the gap related to complex models allowing to retrieve individual parameter estimates and magnitude of variabilities. Its final structure is likely to be applied to clinical data, similar to the works by Ouerdani et al. [48] or Betts et al. [49], in which a strict data-driven model developed in mice led to a simpler and less mechanistic model that could also be applied in the clinical scenario.

## 5. Conclusions

In the current investigation, a semi-mechanistic model to account for the pharmacodynamic effects of three different immuno-therapeutic agents in monotherapy or in combination was developed, incorporating the dynamics of immune cells and their interaction with the tumor. This quantitative analysis has shown that; (1) efficacy can be derived from a combination of agents that show no efficacy as monotherapy, and (2) APC activation is a required component of an effective combination in cold tumors. The model can be further used to leverage parameters components and parameters estimates of recent system pharmacology models, explore the impact of treatment sequences and schedules, and serve as a structural platform for model development in clinical scenarios.

## Figures and Tables

**Table 1:** Summary of study design characteristics.

Variable	Control	Monotherapy			Bi-Therapy			Triple-Therapy
		Ag	PIC	αPD1	Ag and PIC	Ag and αPD1	PIC and αPD1	Ag and PIC and αPD1
Number of animals	27	6	6	12	12	12	12	34
Observations	190	61	56	124	256	241	142	894
% of BQL	39.2	0	0	0	33.2	33.1	0	39.2
Antigen dose (μg)		100			100	100		100
PIC dose (μg)			50		50		50	50
αPD1 dose (μg)				200		200	200	200

E7 long peptide (Antigen (Ag)), polyinosinic-polycytidylic acid (PIC), anti-PD1 (αPD1). Percentage of data below the quantification limit (% BQL).

**Table 2:** Overview of the biological processes considered by the model, together with the different model assumptions and the experimental data supporting those processes.

Biological Processes	Model Assumption	Experimental Data
Exponential tumor growth of unperturbed tumors.	Exponential growth is governed by a parameter (λ) and dependent on TS (Equation (2)) [30,31].	Control (untreated) data was used to estimate unperturbed tumor growth dynamics.
Once the therapeutic agent enters the systemic circulation, it distributes fast and it is eliminated following the first-order rate process [32].	Exponential decay for all the administered treatments (kinetic-pharmacodynamics (K-PD) approach) [32] (Equation (3)) [24,32,33,34].	Absence of drug plasma concentrations for the three different agents.
Activation of APCs by the Ag is needed to trigger a therapeutic effect [1,13,17].	APCs will only be present in the system if the antigen is administered (Equation (4) when i = Ag) [24].	Even though tumor shrinkage is not observed in any of the mice receiving the Ag in monotherapy, there is one that slows tumor progression and can do to the cold nature of the tumor that can be considered as a responder. Tumor shrinkage was only observed in bi-therapy of Ag and PIC, or Ag and αPD1, or triple-therapy (Ag and PIC and αPD1)
APCs trigger the activation and proliferation of naïve CD8 T cells [1,35].	CD8 cells are activated by APCs (Equation (5)) [23].	
Activated CD8 have demonstrated to play an essential role in the antitumor response [1,15].	Tumor cell death is promoted by the activated CD8 T cells (CD8act) (Equation (6)) [23,36,37].	
PIC in combination with an antigen such as E7 long peptide, induces an increase in CD8^+^ T cells [15,38].	Exacerbation and maintenance by a toll-like receptor of the process activated by the antigens (Equations (7) and (8)) [12].	Mice treated with Ag and PIC showed a higher tumor response compared to Ag monotherapy.
Some of the resistance mechanisms developed by tumors include the expression of PD-1 in CD8 cells, regulatory T cells, or MDSCs. The administration of immune checkpoints will inhibit some of these mechanisms, increasing the response to immunotherapy [2,15,18].	Presence of tumor resistance mechanisms used to evade CD8 T cells-mediated death such as the recruitment of immune suppressor cells (e.g., Treg) and expression of the PD-L1 ligand leading to CD8 T cell exhaustion, αPD1 inhibits the tumor resistance mechanisms, which can be at least partly blocked by immune checkpoint inhibitors (Equations (8) and (9)) [21,23,39,40].	After the administration of Ag and αPD1, tumor response is observed in a certain number of mice.

**Table 3:** Model parameter estimates.

Parameter	Estimate (RSE %)	(5th–95th)	IAV (RSE %)	(5th–95th)
TS_0_ (mm^3^)	19.5 (6.88)	(16.03–22.74)	NE	NE
*K_Ag_* (day^−1^)	4.93	NE	NE	NE
λ (day^−1^)	0.194 (3.32)	(0.183–0.209)	0.557 (15.8)	(0.483–0.603)
$\theta_{CD8}$ (au × day^−1^)	1.63 (17.2)	(1.27–2.27)	1.24 (43.2)	(0.607–1.36)
K_PIC_ (day^−1^)	0.721 (42.4)	(0.347–4.194)	3.17 (67.7)	(1.44–953)
θ_PIC_ (au^−1^)	1200 (4.48)	(601.8–1765)	NE	NE
K_αPD1_ (day^−1^)	2.3 × 10^−3^ (79.1)	(6.43 × 10^−5^–9.09 × 10^−3^)	5.16 (42.5)	(1.67–214)
θ_αPD1_ (au^−1^)	821 (2.17)	(548.2–913.3)	NE	NE
Residual error (Log (mm^3^))	0.597 (4.12)	(0.513–0.678)	NE	NE

Results from 1000 bootstrap analysis are shown in round brackets and with the 90% confidence interval. Parameters are defined in the text. Value of ε-shrinkage calculated as 1-sd (IWRES) is 15%. IAV inter-animal variability expressed as coefficient of variation ($\sqrt{e^{\omega^{2}}-1}$), *NE* not estimated, RSE relative standard error.

## Data Availability

The datasets used during the current study are available from the corresponding author on reasonable request.
